# Distinct involvements of the subthalamic nucleus subpopulations in reward-biased decision-making in monkeys

**DOI:** 10.1101/2025.11.04.686631

**Published:** 2025-11-06

**Authors:** Kathryn Branam, Joshua I. Gold, Long Ding

**Affiliations:** Department of Neuroscience, University of Pennsylvania, Philadelphia, PA 19104

## Abstract

The subthalamic nucleus (STN) is a part of the indirect and hyperdirect pathways in the basal ganglia (BG) and has been implicated in movement control, impulsivity, and decision-making. We recently demonstrated that, for perceptual decisions, the STN includes at least three subpopulations of neurons with different decision-related activity patterns (Branam et al., 2024). Here we show that, for decisions that require both perceptual and reward-based processing, many STN neurons are sensitive to both sensory evidence and reward expectations. Within a drift-diffusion framework, STN subpopulations show different relationships to model components reflecting formation of the decision variable, dynamics of the decision bound, and non-decision-related processes. The subpopulations also differ in their representations of quantities related to decision evaluation, including choice accuracy and reward expectation. These results suggest that the STN plays multiple roles in decision formation and evaluation to guide complex decisions that combine multiple sources of information.

## Introduction

The subthalamic nucleus (STN) is a part of the indirect and hyperdirect pathways in the basal ganglia (BG). It provides major glutamatergic inputs to the output nuclei of the BG, namely the substantia nigra pars reticulata (SNr) and the internal segment of the globus pallidus (GPi). Because of its strategic location in these pathways, the STN has been studied extensively in the context of movement control (e.g., Martin, 1927; Martin and Alcock, 1934; Whittier and Mettler, 1949; Carpenter et al., 1950; Wichmann et al., 1994; DeLong and Wichmann, 2001) and is a primary target site for clinical interventions to improve motor functions (e.g., deep brain stimulation in Parkinson’s disease; DeLong and Wichmann, 2001). Functionally, the STN has been linked to inhibitory control of actions or impulsivity (e.g., Baunez et al., 2001; Desbonnet et al., 2004; Witt et al., 2004; Aron and Poldrack, 2006; Frank et al., 2007; Isoda and Hikosaka, 2008; Forstmann et al., 2012; Schmidt et al., 2013; Pasquereau and Turner, 2017; Bonnevie and Zaghloul, 2019).

In parallel to these studies in the motor domain, more recent studies have implicated the STN in a variety of more cognitive functions. These functions include reward-based decision-making (van Wouwe et al., 2011; Espinosa-Parrilla et al., 2015; Seymour et al., 2016; Zénon et al., 2016; Nougaret et al., 2022; Pagnier et al., 2024), noisy evidence-based decision-making (Bogacz and Gurney, 2007; Ratcliff and Frank, 2012; Wei et al., 2015; Zaehle et al., 2017; Branam et al., 2024), and decision conflict resolution (Lehericy et al., 2004; Aron et al., 2007; Fumagalli et al., 2011; Brittain et al., 2012; Zaghloul et al., 2012; Zavala et al., 2017). To support these diverse functions, the STN may contain subpopulations of neurons with distinct computational roles.

Consistent with this idea, we and others previously identified subpopulations of STN neurons with distinct activity patterns when recorded during performance of single tasks (Zavala et al., 2017; Branam et al., 2024). For example, we identified three subpopulations (Branam et al., 2024) that align roughly with three different computational models of STN’s contributions to perceptual decision-making (Bogacz and Gurney, 2007; Ratcliff and Frank, 2012; Wei et al., 2015). However, the specific computational roles that these different subpopulations play in decision-making and other cognitive functions remain not well understood, in part because they have not been examined under a broad enough range of conditions to fully characterize those roles. Here we build on our previous work by recording from individual STN neurons in monkeys performing a more complex decision task that requires integrating noisy evidence accumulation with reward preference. We show that this increased computational task demand can help delineate the diverse computational roles played by different STN subpopulations in the cognitive control of behavior.

## Results

**Figure 1:**
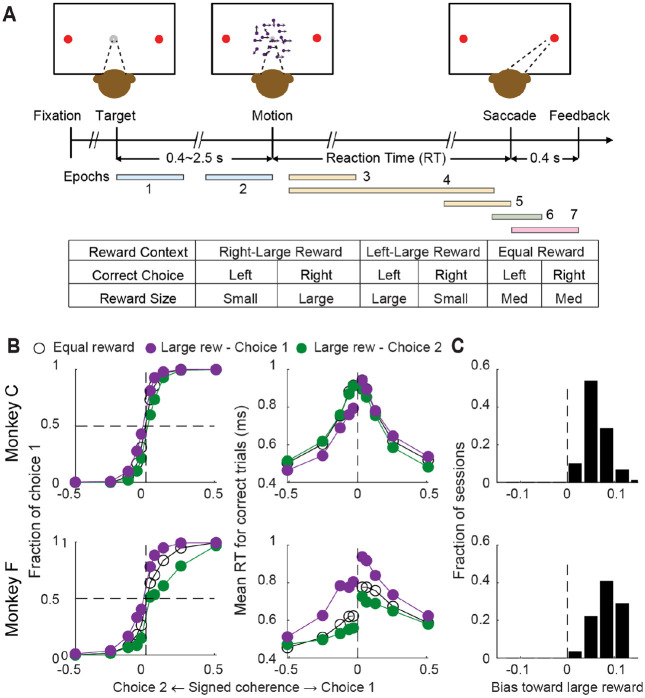
Monkeys biased toward choices associated with large reward A, Task design and timeline. Monkeys made saccades to indicate their perceived motion direction. Correct trials were rewarded based on the reward context (see Table inset). Error trials were not rewarded. “Epochs” illustrate the time windows for epoch-based analyses B, Average choice (left) and RT (right) behavior of the two monkeys. Monkey C: 91 sessions, 37,291 trials; Monkey F: 59 sessions, 25,481 trials. Filled and open circles are data from the three reward contexts, as indicated at the top of the panel. C, Histograms of reward bias for all sessions, estimated using logistic fits to choice data.

We used the same two monkeys as in the previous study of STN neurons (Branam et al., 2024), who were trained to perform a visual motion discrimination decision task by making a saccade at a self-chosen time to indicate their perception of the global motion direction of a random-dot kinematogram (Figure 1A). Here we also used an asymmetric-reward version of the task, for which we separately manipulated the noisy evidence and reward context (a larger juice reward for a correct choice associated with one of the two directions). For each trial, the motion strength and direction were chosen randomly from five values and two directions, respectively. In a block of trials (median: 55 trials, [5 95] percentile: [41 63] trials), one choice was paired with a large reward, and the other was paired with a small reward. The choice-reward association (i.e., “reward context”) was alternated between blocks and signaled to the monkeys at block transitions via changes in the colors of the choice targets. The monkeys were rewarded for correct choices only. The behavioral performance of the monkeys has been documented extensively (Fan et al., 2018; Doi et al., 2020; Fan et al. 2020), with both showing consistent biases toward the large-reward choice (Figure 1B, C).

### STN combines visual and reward information at the single-neuron level

**Figure 2:**
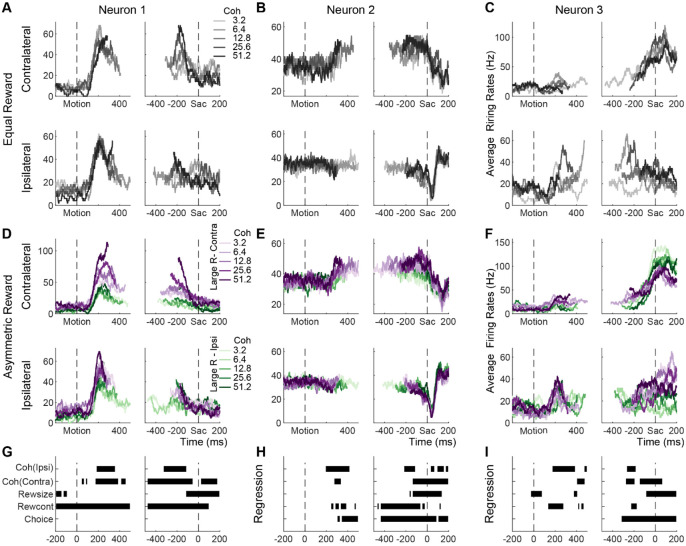
Example STN neurons showing modulation by decision-related factors A-C, Average activity from the equal-reward task, for trials with contralateral (top row) and ipsilateral (bottom row) choices. Activity was truncated at median RT for each trial condition. D-F, Average activity from the asymmetric-reward task, for trials with contralateral (top row) and ipsilateral (bottom row) choices. Activity was truncated at the median RT for each trial condition. Purple and green colors indicate blocks with the large reward paired with contralateral and ipsilateral choices, respectively G-I, Results from a multiple linear regression. Each line shows the timing of significant non-zero coefficients for a specific regressor (t-test, p<0.05). Results for the interaction terms are not shown.

While the monkeys were performing the equal- and asymmetric-reward versions of the task, we measured single-neuron activity of 150 STN neurons (n=91 and 59 for monkeys C and F, respectively). Many single STN neurons were sensitive to manipulations of visual evidence and reward contexts. Figure 2 shows the activity of three example neurons on the equal- (panels A–C) and asymmetric- (panels D–F) reward tasks. The first example neuron showed choice- and coherence-independent activation after motion onset, which dissipated approaching saccades, on the equal-reward task (Figure 2A). On the asymmetric-reward task, this neuron showed similar activation for the two choices but became sensitive to motion coherence (Figure 2D; e.g., in trials with contralateral choices, dark purple curves are higher than light purple curves). The activation also depended on reward context (purple curves are higher than green curves). Multiple linear regression results confirmed these visual impressions (Figure 2G). The second example neuron showed weak choice- and coherence-modulated activation in the late motion viewing period, followed by choice-dependent post-saccade suppression, on the equal-reward task (Figure 2B).

On the asymmetric-reward task, the general choice- and coherence-modulation patterns held, with additional reward-context modulation during motion viewing and reward-size modulation around saccade onset (Figure 2E,H; purple curves are higher than green curves for trials with contralateral choices and the reverse for trials with ipsilateral choices). The third example neuron showed choice-selective activation on the equal-reward task (Figure 2C). On the asymmetric-reward task, this neuron’s activity showed reward-context modulation during early motion viewing, strong choice modulation closer to saccade onset, and reward-size modulation peri- and post-saccades (Figure 2F, I). These example activity patterns highlight the heterogeneity of STN responses, for not only the conversion of visual evidence into decisions as previously shown (Branam et al 2024), but also the incorporation of visual evidence and reward information.

**Figure 3:**
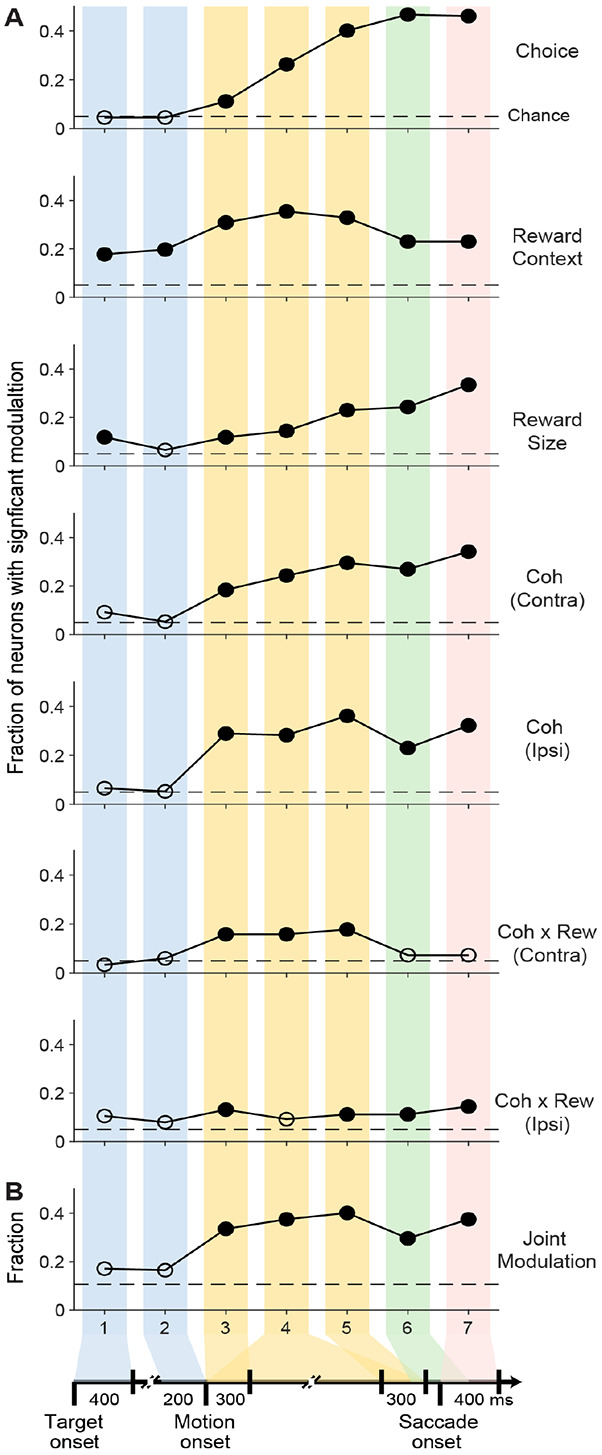
STN activity reflects incorporation of visual evidence and reward information A, Fractions of neurons with significant modulation by task-related factors in the seven task epochs (defined in Figure 1A), as identified by a multiple linear regression (Eq. 2). Horizontal dashed lines: chance levels. Filled circles: fractions that were significantly above chance levels (Chi-square test, p<0.05). B, Fractions of neurons with joint modulation, defined as significant modulation by motion coherence (for either choice) and reward context or reward size, as well as significant modulation by the coherence-reward size interaction terms.

Across the population, substantial fractions of STN neurons showed sensitivity to decision-related factors (Figure 3A). The fraction of neurons with statistically reliable choice modulation (Chi-square test, *p*<0.05) increased steadily during motion viewing (epochs indicated by yellow bands) and plateaued around saccade onset. The fraction for reward context stayed above chance level throughout the trial, with the highest values during motion viewing. The fraction for expected reward size rose above chance level during the later stage of motion viewing and persisted through saccades. The fractions for motion coherence were above chance level after motion onset for trials, with similar values for trials with contralateral or ipsilateral choices. There were also small but significant fractions of neurons that were sensitive to the motion coherence-reward size interaction during motion viewing. Among the neurons with significant modulation, the polarity and timing of modulation varied (Figure 3-S1). Generally, there tended to be more neurons preferring contralateral choices, the context of pairing large rewards with contralateral choices, larger expected reward size, and higher coherences (more purple versus green pixels in the heatmaps). The peak modulation times varied among neurons for all decision-related factors and both polarities.

Using a previous definition of “joint modulation” (Doi et al., 2020), including modulation separately by motion coherence and reward context or reward size and modulation by the interaction of motion coherence and reward size, we found that ~40% of the neurons showed joint modulation during motion viewing and that a substantial amount of joint modulation persisted after saccade onset (Figure 3B). These results are consistent with the idea that the STN contributes to the formation and evaluation of complex decisions that incorporate both visual evidence and reward information, at both the single-neuron and population levels.

The modulation patterns in STN neurons were comparable to those found in the caudate nucleus, another basal ganglia input structure (Figure 3-S2). Compared to a prefrontal region, the frontal eye field (FEF), both the caudate and STN showed less prevalent choice modulation, more prevalent reward context modulation during motion viewing, and more prevalent reward size and coherence modulation around/after saccade onset. These inter-regional differences suggest that the basal ganglia are more directly involved in mediating decisions that require incorporation of visual and reward information.

### STN subpopulations differ in their representations of visual and reward information

To focus on neurons with the most robust task-relevant activity, we inspected the average firing rates of all neurons and identified 66 neurons with the most visible task-related modulations (n = 48 and 18 for monkeys C and F, respectively). The remaining analysis results were based on these neurons.

**Figure 4:**
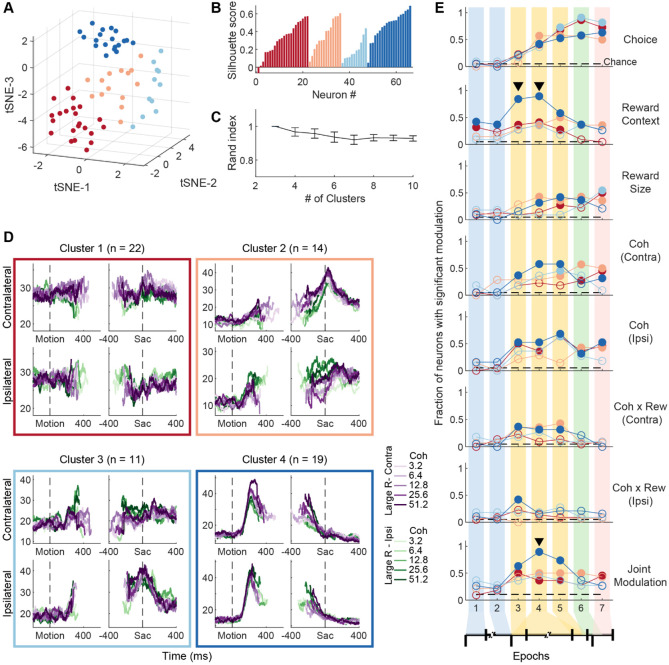
STN consists of subpopulations with distinct activity patterns. A, Visualization of clusters in the tSNE space. Only neurons that passed visual inspection for task-related modulations were included Colors indicate cluster identity and are used in other panels. B, Silhouette scores for neurons C, Rand index values (mean±sd) between multiple iterations of clustering based on different specified cluster numbers. D, Average firing rates for each cluster of neurons for asymmetric-reward trials, separately for trials with contralateral (top) and ipsilateral (bottom) choices and aligned to motion (left) and saccade (right) onsets. Colors indicate coherence levels and reward context (see legend). E, Fractions of neurons with significant modulation by task-related factors in the seven task epochs, separately for each cluster (colors follow those in D. Same format as Figure 3. Horizontal dashed lines: chance levels. Filled circles: fractions that were significantly above chance levels (Chi-square test, p<0.05). Triangles indicate epochs in which there was a significant difference among clusters (Chi-square test, p<0.05/7 epochs).

Using k-means clustering analysis, we grouped these neurons into four clusters, based on 60-dimension activity vectors that contained their z-scored firing rates for two choices, five coherence levels, two reward contexts, and three task epochs. Although the clustering was performed directly on the activity vectors, the clusters also appeared stable and separate in the t-distributed Stochastic Neighbor Embedding (tSNE) space (Figure 4A, Figure 4-S1D). We compared the clustering results from different settings and distance metrics and determined that four or five clusters defined with the cosine metric best described our samples (Figure 4B,C, Figure 4-S1A-C).

Three clusters appeared especially robust regardless of the clustering settings (compare the cluster-average firing rates in Figure 4D and Figure 4-S1E and F). Cluster 2, marked with the orange box, showed a gradual increase in activity during decision formation and was more active for contralateral choices. Cluster 3 (light blue) also showed a gradual increase but was more active for ipsilateral choices. Cluster 4 (dark blue) showed early activation that gradually returned to baseline during motion viewing. Similar average firing rate patterns emerged when the samples were divided into five clusters (Figure 4-S1F). In comparison, the red cluster appeared to include neurons with less defined/stable activity patterns.

Because of the strong, context-dependent modulations of neural activity patterns in STN, clusters differed substantially between those determined with equal-reward and asymmetric-reward data, respectively. For example, as shown in Figure 2, although some features of activity were maintained between the equal- and asymmetric-reward conditions, asymmetric reward conditions could also induce coherence dependence (e.g. Neuron 1, compare activity aligned to motion onset for contralateral choices) or changes in temporal dynamics (e.g., Neuron 3, compare peri-saccade activity for ipsilateral choices), in addition to introducing novel reward context modulation patterns. These differences in activity features had substantial effects on how the neurons were clustered: the Rand index values between clustering using equal- and asymmetric-reward activity data were much lower than those computed within reward conditions (Figure 4-S1G), indicating weak correspondence between the two clustering methods. The following analyses focused on the four clusters identified using the asymmetric-reward data.

These clusters showed different sensitivities to several decision-related factors from the asymmetric-reward task (Figure 4E). All clusters showed similar gradual emergence of choice sensitivity during motion viewing. Cluster 1 (red) showed reward context modulation that emerged before motion onset and disappeared before saccade onset, reward size modulation around saccade onset, and coherence modulation only for trials with ipsilateral choices. Cluster 2 (orange) showed reward context and size modulation that emerged later during motion viewing but persisted through saccades, coherence modulation for both choices in late motion viewing and peri-saccade epochs, and sensitivity to coherence-reward interaction for contralateral choices during all three motion viewing epochs (epochs 3-5). Cluster 3 (light blue) did not show above-chance prevalence of reward context modulation, was sensitive to reward size only after saccade onset, and sensitive to coherence only during late motion viewing (epoch 5). Cluster 4 (dark blue) showed persistent reward context modulation almost throughout the trial (except for the post-saccade epoch 7), reward size modulation during late motion viewing and pre-saccade period (epochs 4-6), and coherence modulation for both choices during motion viewing and saccades. The prevalence of reward context modulation was especially high for Cluster 4 during motion viewing, contributing to a higher prevalence of joint modulation than for other clusters (indicated by triangles). The prevalence of joint modulation was comparable for Clusters 1 and 2 but was not above chance for Cluster 3. These different patterns of decision-related sensitivities suggest that the STN subpopulations may play distinct roles in decision-related computations.

### STN subpopulations differ in their relationship to decision formation-related computational components

**Figure 5:**
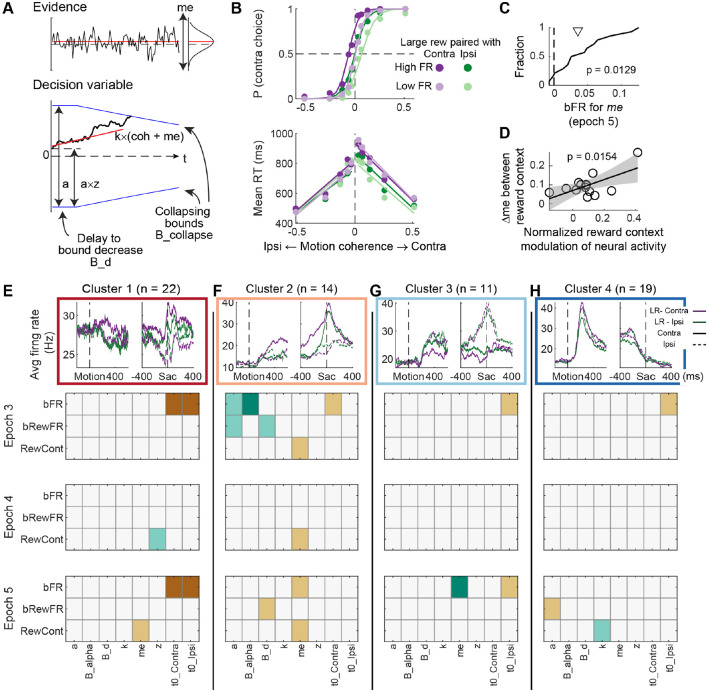
STN subpopulations relate differently to computational components in a DDM. A, Illustration of the DDM. B, Example of average choice and RT performance for trials split by firing rates in Epoch 5 of neurons in the second cluster. Circles performance for trials grouped by firing rates and reward contexts. Curves: fits by logistic (choice) and linear (RT) functions. C, Cumulative density function of regression coefficient (bFR) for the fitted me parameter from behavioral data in B. Triangle: median value. Raw p value: sign test. D, Scatterplot of the difference in me between reward contexts (the large reward was paired with contralateral or ipsilateral choices) and the regression coefficient for reward context of neural activity measured in Epoch 5 (normalized by average activity across conditions, i.e., b0 term in the regression). Data are from neurons in the second cluster. Raw p value: t-test. E-H: Relationship between activity measured in the three epochs and DDM components for the four clusters identified in Figure 4. Top row shows simplified plots of average firing rates, pooling across coherence levels. Colors indicate different reward contexts. Solid and dashed lines indicate trials with contralateral and ipsilateral choices, respectively. The next three rows show results based on activity measured in epochs 3-5 (during decision formation). For each matrix panel, the top row: sign test results for the null hypothesis that the regression coefficients for the effect of firing rate on the DDM component have a zero median (e g.,see C). Second row: sign test results for the null hypothesis that the regression coefficients for the reward context-firing rate interaction have a zero median. Third row: Pearson correlation results for the null hypothesis that the reward context modulation of neural activity is not correlated with the difference in the DDM component between reward contexts (e.g., see D). Dark brown/teal positive/negative median with a p<0.05/8 (DDM components)/3 (epochs). Light brown/teal: positive/negative median with a p<0.05.

To better understand how the activity patterns of STN neurons related to the computations used to form the decision, we analyzed the behavioral and neural data using a drift-diffusion model (DDM; Figure 5A). The DDM quantifies decision formation in terms of the (possibly biased) accumulation of noisy sensory evidence to a predefined bound that governs choice and RT, which accounts well for behavioral performance on this task (Fan et al., 2018). To probe the behavior-neural relationship, we performed two sets of analysis. First, we split the trials within a session by a given neuron’s median firing rate during a given task epoch, separately for the two reward contexts, resulting in trials with high and low firing rates for each reward context (e.g., Figure 5B). The full set of average choice and RT results from splitting trials based on firing rates for different clusters and task epochs are shown in Figure 5-S1. We fitted the DDM separately to trials with each firing rate-reward context combination from each individual session and performed a regression analysis to quantify the dependence of each DDM parameter on the high-low firing rate category and firing rate-reward combination interaction. We assessed whether these regression coefficients exhibited consistent trends within a cluster. For example, Figure 5C shows the cumulative density function of the regression coefficient for firing rate (bFR) from epoch 5 for the parameter *me*, indicating a trend of observing higher firing rates when there was a greater bias in momentary evidence (i.e., the value of this regression coefficient from most sessions was >0) , regardless of reward context.

Second, we measured the Pearson’s correlation coefficient between the difference in a given DDM parameter between reward contexts and the relative extent of reward context modulation of neural activity (measured in a specific epoch) in the same sessions (i.e., $\beta_{RewCont}∕\beta_{0}$ in Eq. 7. The correlation was performed separately for each cluster. For example, Figure 5D shows the scatterplot of the difference in *me* and reward context modulation of neural activity in a cluster and the significant correlation suggests that this cluster may contribute to reward context-biased adjustment in *me*.

These two sets of analysis indicated distinct relationships between STN neural activity in each of the subpopulations and behaviorally derived computational components of the decision process. Figure 5E summarizes the results from the two sets of analysis for all four clusters. The cumulative density functions and scatterplots associated with these summaries are shown in Figure 5-S2. For simplicity, cluster identity was represented with a more compact depiction of the average activity patterns by pooling across all coherence levels.

Cluster 1 neurons had activity patterns that were consistent with controlling non-decision processes and mediating the reward biases in *me* and *z*. Specifically, their firing rates in epochs 3 and 5 were positively related to the non-decision times for both contra- and ipsi-lateral choices (Figure 5E, dark brown pixels for bFR vs. *t0_Contra* and *t0_Ipsi*), regardless of reward context, suggesting that higher activity in this cluster reflects longer time for general visual and/or motor processing. When the full motion viewing period was considered (epoch 4), greater reward context modulation of this cluster’s activity tended to reflect a reduction in reward context-modulated estimates of *z* (light teal pixel, RewCont vs. z). When only the late motion viewing epoch was considered (epoch 5), greater reward context modulation of this cluster’s activity tended to reflect a reduction in reward context-modulated estimates of *me* (light teal pixel, RewCont vs. *me*).

Cluster 2 neurons appeared to be more consistently involved in computational components related to decision bounds and reward bias in *me* (Figure 5F). Their activity in early motion viewing (epoch 3) was negatively associated with the total bound height and the collapse rate in a reward-independent manner (bFR vs. *a* and *B_alpha*); i.e., higher activity was associated with a lowering of total bound and slower collapsing. The activity-reward context interaction was also negatively associated with the total bound height and the delay in bound collapse (bRewFR vs. *a* and *B_d*), suggesting that the reduction was more prominent when contralateral choices were paired with the large reward. The association with the delay in bound collapse reversed signs in the late motion viewing epoch (epoch 5). Beyond these bound-related relationships, higher activity of this cluster in late motion viewing also reflected a larger *me* bias toward the contralateral choice (bFR vs. *me*). In addition, across all epochs during motion viewing, the reward context modulation in this cluster’s activity was positively correlated with the reward bias in *me* (RewCont vs. *me*), suggesting that this cluster may contribute to mediating this specific form of bias in both reward-dependent and independent manners.

The remaining two clusters appeared to have more limited relationships with decision formation. Both clusters showed a positive relationship between firing rates and non-decision times for the ipsilateral choice (bFR vs. *t0 Ipsi*). Cluster 3 showed a negative relationship between its activity during late motion viewing and *me,* independent of reward context (bFR vs. *me*), suggesting that higher activity reflected a larger bias toward the ipsilateral choice. This relationship had the opposite laterality as that for the Cluster 2: for Cluster 2, higher activity in the contralateral-preferring neurons reflected a larger bias toward the contralateral choice, whereas for Cluster 3, higher activity in the ipsilateral-preferring neurons reflected a larger bias toward the ipsilateral choice. Cluster 4 showed a negative correlation between its reward context modulation and reward context-dependent changes in *k* (RewCont vs. *k*), suggesting that the rate of decrease in this cluster’s activity reflected the rate of evidence accumulation. In addition, higher activity in the cluster during late motion viewing reflected higher total bound height when the large reward was paired with contralateral choices (epoch 5, bRewFR vs. *a*).

These observed effects were robust with different ways of clustering (Figure 5-S3). For example, the cluster with a contralateral preference (Cluster 2, orange) was consistently related to bound-related parameters and reward context modulation of *me* (cluster 3 in Figure 5-S3A and cluster 5 in Figure 5-S3B). The cluster with an ipsilateral preference (Cluster 3, light-blue) was consistently related to ipsilateral non-decision time and *me* (cluster 1 in Figure 5-S3B). The cluster with early activation (Cluster 4, dark blue) consistently showed a negative correlation between its activity in epoch 5 and parameter *k* (cluster 1 in Figure 5-S3A, when three clusters were used, and cluster 3 in Figure 5-S3B, when five clusters were used).

Taken together, these results suggest that the STN as a whole may contribute to decision and non-decision (e.g., basic visual and/or motor) processes, with different subpopulations playing different roles. The two clusters with more prominent choice preferences (Clusters 2 and 3) may be involved in mediating the bias in momentary evidence in opposite directions. Compared to other clusters, Cluster 2 was more closely related to bound-related components, in reward context-dependent and independent manners, and more consistently related to reward bias in *me.* Cluster 1 was most consistently involved in non-decision processes. Cluster 4 may be involved in reward context-dependent modulation of bound height (*a*) and the evidence scaler *k.*

### STN subpopulations differ in their representations of decision evaluation-related signals

**Figure 6:**
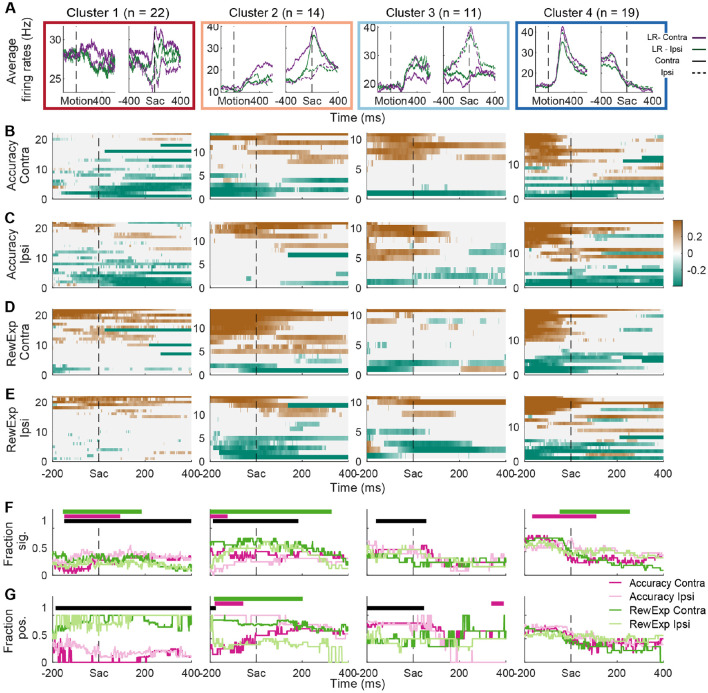
STN subpopulations relate to decision evaluation signals. A, Simplified plots of average firing rates, pooling across coherence levels, for the four clusters of neurons. Same as Figure 5E. B,C, Heatmaps of partial correlation coefficients between neural activity and post-decision choice accuracy for contralateral (B) and ipsilateral (C) choices, after accounting for the effects of reward expectation. Each column shows the results from one neuron cluster. D,E, Heatmaps of partial correlation coefficients between neural activity and post-decision reward expectation for contralateral (D) and ipsilateral (E) choices, after accounting for the effects of choice accuracy . F, Fractions of neurons with non-zero correlation coefficients (t-test, p<0.05). Each column shows the results for one neuron cluster. Colors indicate different evaluation signals. Horizontal black bars on top indicate timepoints when the fraction values significantly differed between accuracy and reward expectation (Chi-square test, p<0.05). Pink and green bars indicate timepoints when the fraction values significantly differed between choices for accuracy and reward expectation, respectively. G, Fractions of neurons with positive correlation coefficients. For each time point, only neurons with non-zero correlation coefficients were included. Same format as F.

The different STN subpopulations also exhibited different patterns of responses with respect to different forms of decision evaluation. As demonstrated previously, the asymmetric-reward manipulation partially decorrelates choice accuracy and reward expectation (Fan et al., 2024). Following our previous approaches, we computed the partial Spearman correlation between average activity in a given time window and each of the two quantities (choice accuracy and reward expectation), while controlling for the other (Figure 6).

In Cluster 1, a larger fraction of neurons represented choice accuracy than reward expectation (Figure 6B-F, first column). For choice accuracy, the fraction was higher for ipsilateral choices (Figure 6F, light pink above pink). For reward expectation, the fraction was higher for contralateral choices (green above light green). The dominant signs of representation differed between evaluation signals: higher activity tended to be associated with lower choice accuracy (Figure 6B,C, more teal pixels) and higher reward expectation (Figure 6D,E, more brown pixels; quantification in Figure 6G).

In Cluster 2, a larger fraction of neurons represented reward expectation than choice accuracy (Figure 6, second column; F: green/light green over pink/light pink curves). For reward expectation, the fraction was higher for contralateral choices (Figure 6F, second column, green over light green). The dominant signs of representation differed between evaluation signals and choices: higher activity tended to be associated with higher and lower reward expectation for contralateral and ipsilateral choices, respectively, whereas the reverse was true for choice accuracy.

In Cluster 3, a larger fraction of neurons represented choice accuracy than reward expectation, without additional choice selectivity. The dominant signs of representation differed between evaluation signals but not choices: higher activity tended to be associated with higher choice accuracy and lower reward expectation, for both choices.

In Cluster 4, the fractions of neurons with evaluation signal representations differed between task timing and choices but not between choice accuracy and reward expectation. The fractions were higher before than after saccade onset. The choice selectivity also switched around saccade onset: more neurons represented evaluation of contralateral choices before saccade onset and ipsilateral choices after saccade onset.

## Discussion

Using an asymmetric-reward random-dot visual motion discrimination task, we showed that neurons in the monkey STN have context-dependent activity patterns that encode various components of decision formation and evaluation. The neurons can be divided into at least 4 subpopulations that may support different functions, assessed in the context of an accumulate-to-bound modeling framework. Cluster 1 neurons encode signals related to non-decision times and the evaluation of choice-dependent accuracy. Cluster 2 neurons encode signals related to reward context-dependent modulation of decision bound dynamics, contralateral biases, and choice-dependent reward expectations. Cluster 3 neurons encode signals related to ipsilateral biases and the evaluation of choice-independent accuracy. Cluster 4 neurons encode signals related to reward context-dependent changes in the decision bound, the scaling of visual evidence, and choice-dependent evaluation based on both accuracy and reward expectation.

These results support and extend our previous study of STN activity in the same monkeys performing the symmetric-reward version of the motion discrimination task. For example, the average activity of the present Cluster 2 appears similar to that of cluster 1 from the previous study, both showing slow ramping activity during motion viewing and prominent choice-dependent peri-saccade activity. We previously hypothesized that this subpopulation may be more closely linked to the evidence-accumulation-to-bound process than the other subpopulations. Our present results provide further support to this hypothesis in the context of reward-biased evidence accumulation, with the activity of this subpopulation linked to bound dynamics and reward-biased momentary evidence.

Moreover, the average activity of the present cluster 4 appears similar to that of cluster 2 from the previous study, both showing early activation that gradually dissipated during motion viewing. We previously hypothesized that this subpopulation may contribute to controlling the collapsing bound or urgency signal, based on the temporal trajectory of its activity. Contrary to this hypothesis, we did not detect any consistent relationship between neural activity and DDM parameters that control the bound dynamics (i.e., *B_alpha* or *B_d*). Instead, we found relationships between its reward context-modulated neural activity and the total bound height (*a*) and the scaling factor for evidence (*k*). The interpretation of these relationships, however, has a caveat: they were observed only during the late motion viewing period (Epoch 5), when the neural activation was largely diminished. The functional roles of this subpopulation remain elusive and especially intriguing because of the observed paradox: activity of this subpopulation was much more likely than those of other subpopulations to depend on reward context during decision formation (Figure 4E), but the activity and its reward context modulation did not appear to be strongly related to any decision formation-related behavioral measures that we probed.

The prevalence of joint modulation by reward and evidence-related factors in our sample was comparable to what we observed in the caudate (Figure 3-S2; Doi et al., 2020; Fan et al., 2020), suggesting that the reward-biased decision behavior reflects interactions among multiple BG nuclei (and likely other brain regions). Both the caudate nucleus and STN receive dopaminergic innervation from the substantia nigra pars compacta (Galvan et al., 2014), which may carry reward-related signals, and cortical projections, which may carry evidence accumulation-related signals (Bogacz and Gurney, 2007; Ding and Gold, 2012a; Ding, 2015; Wei et al., 2015). These inputs may be combined in the STN to support the generation of a common-currency decision variable. Alternatively, it is possible that the STN receives already combined signals either directly from the cortex or indirectly from the caudate projection to the external segment of globus pallidus, which has strong reciprocal connections to the STN. Future work is needed to understand how different BG nuclei obtain decision-related signals that reflect incorporation of reward information and noisy evidence accumulation. Furthermore, both caudate and STN outputs are sent to the output BG nucleus, specifically, the SNr for oculomotor decisions. It remains an open question how the various forms of decision-related signals from multiple BG sources are combined in the BG output to affect the eventual decisions.

Our sample was selected based on neural activity on the visual motion discrimination task. The high fraction of neurons showing joint modulation by reward and noisy sensory evidence in this sample suggested that these STN neurons may participate in a variety of decisions that involve either or both of these factors. It remains to be tested whether the same STN neurons are also involved in decisions or movement modulation based on reward properties alone (Zaghloul et al., 2012, 2012; Espinosa-Parrilla et al., 2015; Zénon et al., 2016; Nougaret et al., 2022). The early-onset activity profile of cluster 4 neurons is particularly intriguing for its possible correspondence to previously identified STN neurons that may mediate response inhibition in stop-signal (countermanding) tasks (Schmidt et al., 2013; Pasquereau and Turner, 2017) or STN neurons that may mediate switching from automated to controlled actions (Isoda and Hikosaka, 2008).

In summary, we characterized single-neuron activity in the STN of monkeys performing a decision task that requires incorporation of noisy visual evidence and different reward context information. These results advanced our understanding of the roles of the STN in decision making, by refining previous hypotheses about its role in the evidence-accumulation-to-bound framework and, for the first time, revealing how activity of different STN subpopulations were modulated to contribute to the formation and evaluation of reward-biased decisions.

## Methods

We used two adult male rhesus monkeys (*Macaca mulatta*) that were trained extensively on the asymmetric-reward visual motion direction-discrimination (dots) task. All training, surgery, and experimental procedures were in accordance with the National Institutes of Health Guide for the Care and Use of Laboratory Animals and were approved by the University of Pennsylvania Institutional Animal Care and Use Committee (protocol # 804726).

### Task design

The behavioral task (Figure 1A) has been described in detail previously (Fan, 2018). Briefly, we trained the monkey to report the perceived motion direction of the random-dot stimulus with a saccade at a self-determined time. The monkey’s choice of saccade was rewarded if it was congruent with the motion direction. The monkey’s eye position was monitored with a video-based eye tracker and feedback for a fixation break or choice error was given based on online comparisons between the monkey’s eye position and task-relevant locations. Motion directions and five levels of motion coherence (defined as the fraction of dots moving coherently in the same direction) were randomized across trials. For the equal-reward dots task, all correct saccades were rewarded with the same medium amount of juice. For the asymmetric-reward dots task, the saccade-reward associations (reward contexts) were alternated in blocks of trials, such that in a given block, a correct saccade to one choice target was paired with a large amount of juice and a correct saccade to the other target was paired with a small amount of juice. Block changes were signaled to the monkey with color cues.

### Electrophysiology

The general surgical and data-acquisition methods were described in detail previously (Ding and Gold, 2010, 2012b). Neural activity was recorded using glass-coated tungsten electrodes (Alpha-Omega) or polyamide-coated tungsten electrodes (FHC, Inc.), driven by a NAN microdrive (NAN Instruments, LTD) mounted on a grid system. STN was localized using the same criteria as described previously (Branam 2024), by comparing MRI scan images, recording coordinates, and nearby landmark locations (thalamus, reticular nucleus of the thalamus, zone incerta, and SNr) and by assessing neurons’ baseline firing patterns.

### Initial data processing and screening

Saccade reaction time (RT) was measured offline with established velocity and acceleration criteria. Trials were excluded if the online and offline detection of choice-indicating saccades were mismatched (e.g., the saccade did not follow the stereotypical trajectory). Single neurons were identified by offline spike sorting (Offline Sorter, Plexon, Inc.). Neurons with fewer than five correct trials per choice × coherence × reward context combination were excluded.

In our previous study of STN activity using liberal inclusion criteria, we identified at least four subpopulations of STN neurons with different activity patterns on the equal-reward dots task, with many cells not displaying any task-related modulation and therefore unlikely involved in the decision process (Branam et al., 2024). In this study, we aimed to focus on neurons with more robust task-related activity. Toward this aim, we visually inspected each neuron’s average firing rates for different choice × coherence combinations. Only neurons with visually apparent task-related modulation were included for more in-depth analysis (i.e., results presented in Figures 5 and 6).

### Behavioral analysis

To quantify reward context-induced biases, a logistic function was fitted to the choice data for all trials for each session:

(Eq. 1) $$P_{contra\;choice}=\frac{1}{1+e^{-Slope(Coh+Bias)}}\;\;,$$

where Coh is the signed motion coherence,

$$Slope=slope0+slope_{rew}\times RewCont,\;Bias=bias0+bias_{rew}\times RewCont,\;RewCont=\{1\;for\;contralateral-large\;reward\;blocks,\;\;-1\;for\;ipsilateral-large\;reward\;blocks\}.$$

To quantify decision-related computations that account for both choice and RT, we fitted a drift-diffusion model (DDM; Figure 5A) to these data simultaneously, following previously established procedures (Fan et al., 2018). Briefly, the DDM assumes that motion evidence is accumulated over time into a decision variable (DV), which is compared to two choice bounds that decrease in magnitude (“collapse”) over time within a trial. A choice is made when the DV crosses either bound, such that the time of crossing determines the decision time and the identity of the bound determines the choice identity. The model has eight basic parameters (presented here in six groups): 1) *a,* the maximal bound height; 2) *B_collapse* and *B_d*, the decay speed and onset specifying the time course of the bound “collapse”; 3) *k,* a scale factor governing the rate of evidence accumulation; 4) *me,* an offset specifying a bias in the rate of evidence accumulation; 5) *z,* an offset specifying a bias in the DV, or equivalently, asymmetric offsets of equal magnitude for the two choice bounds; and 6) *t0_Contra* and *t0_Ipsi,* non-decision times for the two choices that capture RT components that do not depend on evidence accumulation (e.g., visual latency and motor delay). DDM model fitting was performed separately for each session, using the maximum *a posteriori* estimate method (python v3.5.1, pymc 2.3.6) and prior distributions suitable for human and monkey subjects (Wiecki et al., 2013). We performed at least five runs for each variant and used the run with the highest likelihood for further analyses.

To quantify the expected choice accuracy and reward expectation, we used previous methods (Fan, 2024). Specifically, we defined choice accuracy as the estimated accuracy, on average, given the current choice and decision time (DT), as following:

(Eq. 2) Choiceaccuracy={P(Correct∣Right,DT),RighttargetischosenatDTP(Correct∣Left,DT),LefttargetischosenatDT}

where DT is the decision time that equals RT minus non-decision time (estimated from DDM fits). The righthand side was computed by marginalizing over all possible coherences. For example, for Right choices,

(Eq. 3) $$P(Correct|Right,DT)=\sum_{Coh_{i}}[P(Correct|Right,DT,Coh_{i})P(Coh_{i}|Right,DT)]\;=\sum_{Coh_{i}}\frac{P(Correct|Right,DT,Coh_{i})P(Right,DT|Coh_{i})P(Coh_{i})}{\sum_{Coh_{i}}[P(Right,DT|Coh_{i})P(Coh_{i})]}$$

By task design,

(Eq. 4) P(Correct∣Right,DT,Cohi)={1ifCohi>00ifCohi<0}

$P(Right,DT|Coh_{i})$ was obtained by numerical simulation of the DDM using the best-fitting parameters. For each coherence, we obtained the probability of the decision variable (DV) attaining a value x at time t, $pdf_{DV}(t)=P(DV(t)=x|Coh_{i})$, using the best fitting DDM parameters of each session and reward context.


(Eq. 5) $$P(Right,DT|Coh_{i})=\int_{upper\;bound}^{\infty }P(DV(t)=x|Coh_{i})dx\;P(Left,\;DT|Coh_{i})=\int_{-\infty }^{lower\;bound}P(DV(t)=x|Coh_{i})dx$$

After obtaining an estimate of choice accuracy,

(Eq. 6) RewardExpectation=Accuracy×RewardsizeassociatedwiththechoiceRewardsize={1ifsmallrewardlarge∕smalliflargereward}

### Neural activity analysis

To relate activity to decision-related factors, we performed two regression analyses. First, for each single unit, we computed the average firing rates in seven task epochs (Figure 1A): 1) a 400 ms window beginning at target onset (focusing on visual response to target presentation), 2) a 200 ms window ending at dots onset (focusing on activity before motion viewing), 3) a 300 ms window beginning at 100 ms after dots onset (focusing on early motion viewing), 4) a variable-duration window from 100 ms after motion onset to 100 ms before saccade onset (focusing on the whole motion viewing period), 5) a 300 ms window ending at saccade onset (late motion viewing, pre-saccade activity), 6) a 300 ms window beginning at 100 ms prior to saccade onset (peri-saccade activity), and 7) a 400 ms window beginning at saccade onset (post-saccade activity). We performed a multiple linear regression on the spike counts from correct trials, for each task epoch separately.

(Eq. 7) $$Spike\;count=\beta_{0}+\beta_{Choice}\times I_{Choice}+\beta_{RewCont}\times I_{RewCont}+\beta_{RewSize}\times I_{RewSize}\;\;+\beta_{Coh-Contra}\times I_{Coh-Contra}+\beta_{Coh-Ipsi}\times I_{Coh-Ipsi}\;+\beta_{RewCoh-Contra}\times I_{Coh-Contra}\times I_{RewSize}+\beta_{RewCoh-Ipsi}\times I_{Coh-Ipsi}\times I_{RewSize},$$

where

IChoice={1contralateralchoice−1ipsilateralchoice}IRewCont={1contralateral−largereward−1ipsilateralchoice−largereward}IRewSize={1largerewardisexpected−1smallrewardisexpected}ICoh−Conta={cohcontralateralchoice0ipsilateralchoice}ICoh−Ipsi={0contralateralchoiceCohipsilateralchoice}

Significance of non-zero coefficients was assessed using *t*-test (criterion: *p* = 0.05).

Second, for each single unit, we also performed running regressions using Eq. 7 on the spike counts within 150 ms windows with 10-ms steps. These running regressions were performed on activity aligned to target, motion, and saccade onsets separately. Only correct trials were included. Time windows with <10 correct trials were excluded.

### Cluster analysis

We converted each neuron’s activity into a 60-D vector consisting of the average firing rates within three 200-ms windows for all trial conditions. The time windows were during early motion viewing (100-300 ms after motion onset), during late motion viewing (300-500 ms after motion onset), and around saccade (200 ms centered at saccade onset). Twenty trial conditions were combinations of two choices, five coherence levels, and two reward contexts. The firing rates were z-scored by baseline activity measured in a 200-ms window ending at motion onset.

We performed clustering on these vectors using different settings. The variations include 1) using either the full data set (150 neurons) or those that passed visual inspection (66 “good” neurons); 2) specifying the number of clusters from 3 to 10; and 3) using cosine, correlation, or squared Euclidean distance as the distance metric. For each variation, clustering was run 50 times, with each run finding the best clusters from 50 sets of initial centroid positions. For each run, silhouette scores were computed to document the mean score value and the number of negative scores (a positive score indicates that the member is closer to its same-cluster neighbors than different-cluster neighbors). For each variation, Rand index was computed to document the stability of clustering between two runs (a high value indicates that if a pair of members are placed in the same (different) clusters in one run, they are highly likely to be included in the same (different) clusters in another run). Based on the silhouette scores (higher mean, fewer negative values), Rand index (higher mean), and the distribution of units in tSNE space (Figure 4-S1A-D), we determined that separating the datasets into 4~5 clusters using the cosine metric was the most appropriate. The Rand indices for the 66 “good” neurons between clustering from the larger dataset or themselves were above 0.84, indicating a high level of correspondence between the two method variations (Figure 4-S1G). When only units from clusters 2~4 in Figure 4 were considered, the Rand index between the two methods was 0.95, indicating a near-complete match. Therefore, we showed only results based on clustering the “good” neurons.

### Relate neural activity to DDM components

We examined the relationship between neural activity and DDM components in two tests.

First, for each neuron and reward context, we split the trials by the median firing rates in one of the three epochs that covered the motion viewing period (i.e., Epochs 3–5). Average choice and RT performance after the splits are shown in Figure 5-S1 (for these plots, the curves are logistic function fits to choices and linear function fits to RTs). We fitted the DDM separately to trials with different high/low firing rates × reward context combinations. We decomposed the influences of reward context, firing rates, and their interactions on each DDM parameter using the regression:

(Eq. 8) $$Parameter=b_{0}+\text{bRew}\times I_{RewCont}+\text{bFR}\times I_{FR}+\text{bRewFR}\times I_{RewCont}\times I_{FR}$$

where

IFR={1FR>medianvalueforthegivenrewardcontext−1FR<medianforthegivenrewardcontext}IRewCont={1highermedianFR−1lowermedianFR}

We used the sign test to test whether median values of regression coefficients differed from zero for each cluster.

Second, for each neuron, we normalized the regression coefficient $\beta_{RewCont}$ by $\beta_{0}$ in Eq. 7 as the neural index of reward context modulation. For the same session, we computed the difference in DDM parameters between reward contexts as the behavioral index of reward context modulation. For each cluster, we performed Pearson correlation to test whether the two indices were correlated for a given DDM parameter.

### Neural activity analysis: relating activity to decision-evaluation signals

To relate a neuron’s activity to decision-evaluation signals, for each trial, we computed the choice accuracy and reward expectation and measured the firing rates in running 300-ms time windows with 1-ms steps. Only correct trials were included. For each time window, we performed two partial (Spearman) correlations: 1) between firing rates and choice accuracy while removing the effect of reward expectation, and 2) between firing rates and reward expectation while removing the effect of choice accuracy. Significance was assessed at *p* = 0.05. Chi-square tests were performed to compare fractions of significant/positive correlation at each time window.

## Supplementary Material

1 Figure 3-S1: Summary of temporal profiles of modulation of STN activity by decision-related factors.
For each regression factor, heatmaps of significant regression coefficients are plotted in the first row for activity aligned to motion (left) and saccade (right) onsets. Neurons were sorted by the timing of peak magnitude of modulation. The fraction of neurons with significant non-zero coefficients were plotted in the second row. Significance for a coefficient was assessed using t-test (p<0.05). For the fraction plots, the dashed horizontal lines represent chance level. Time bins with values that were significantly above chance (chi-square test, p<0.05) were indicated via thicker lines.
Figure 3-S2: Comparison between STN, FEF and caudate neurons
Each panel shows the fractions of neurons in the three regions (see legend for colors) with significant coefficients (t-test, p<0.05) for a specific regressor (rows) and activity alignment (columns). Horizontal bars indicate results of chi-square tests using a criterion of p<0.05/3(alignments)/7(regressors). Black horizontal bars indicate significant difference between FEF and STN populations. Red horizontal bars indicate significant difference between caudate and STN populations. FEF and caudate data are from Fan, et al. eLife, 2020. N = 126, 136, and 150 neurons for FEF, caudate, and STN, respectively.
Figure 4-S1: Stability of clustering results
A, Silhouette scores for different combinations of distance metrics and number of clusters. Red triangle indicates the clustering presented in Figure 4. Mean and s.d. values were computed from 50 iterations of clustering.
B, Fraction of negative silhouette scores. Same format as A.
C, Rand index. Same format as A.
D, Visualization of clusters from Figure 4 in the tSNE space calculated from all units. Non-gray circles indicate neurons that passed visual inspection. Colors indicate cluster identities that are used in Figure 4.
E, Average firing rates for each cluster of neurons, with the assumption of three clusters. Same format as Figure 4D. Colored boxes indicate clusters that loosely correspond to those in Figure 4.
F, Average firing rates for each cluster of neurons, with the assumption of five clusters. Same format as Figure E.
G, Pairwise Rand index values for clustering based on all or only good units, using asymmetric (AR) or equal-reward (ER) data, and assuming 4 or 5 clusters.
Figure 5 S1 Average choice and RT performance for trials split by firing rates for different neuron clusters and epochs.
Same format as Figure 5B. The columns correspond to the same clusters in Figure 5E.
Figure 5 S2 Raw plots corresponding to the summary in Figure 5E
Top section: average firing rates for the four clusters. Same as the top row of Figure 5E.
Second section: Cumulative density functions of regression coefficient (bFR) for DDM parameters. The title for each plot indicates the DDM parameter and the epoch from which firing rates were used to split the trials. Same format as Figure 5C.
Third section: Cumulative density functions of regression coefficient (bRewFR). Same format as the second section.
Bottom section: Scatterplots of the difference in DDM parameters between reward contexts and the regression coefficient for reward context of neural activity. Same format as Figure 5D.
For the last three sections, only relationships with a p<0.05 from sign test (for bFR and bRewFR) or significant Pearson correlation are shown, corresponding to the colored pixels in Figure 5E.
Figure 5 S3 Assessing the robustness of the regression and correlation results with different clustering settings.
A, Regression and correlation results based on dividing the neurons into three clusters. Same format as Figure 5E.
B, Regression and correlation results based on dividing the neurons into five clusters. Same format as Figure 5E.
