# Supplementary material for: Distinct involvements of the subthalamic nucleus subpopulations in reward-biased decision-making in monkeys
Source: bioRxiv. 2025 Nov 6:2025.11.04.686631. Preprint. [Version 1] doi: 10.1101/2025.11.04.686631 (PMC12637535; doi:10.1101/2025.11.04.686631)
Supplement: 1 — Figure 3-S1: Summary of temporal profiles of modulation of STN activity by decision-related factors. For each regression factor, heatmaps of significant regression coefficients are plotted in the first row for activity aligned to motion (left) and saccade (right) onsets. Neurons were sorted by the timing of peak magnitude of modulation. The fraction of neurons with significant non-zero coefficients were plotted in the second row. Significance for a coefficient was assessed using t-test (p<0.05). For the fraction plots, the dashed horizontal lines represent chance level. Time bins with values that were significantly above chance (chi-square test, p<0.05) were indicated via thicker lines. Figure 3-S2: Comparison between STN, FEF and caudate neurons Each panel shows the fractions of neurons in the three regions (see legend for colors) with significant coefficients (t-test, p<0.05) for a specific regressor (rows) and activity alignment (columns). Horizontal bars indicate results of chi-square tests using a criterion of p<0.05/3(alignments)/7(regressors). Black horizontal bars indicate significant difference between FEF and STN populations. Red horizontal bars indicate significant difference between caudate and STN populations. FEF and caudate data are from Fan, et al. eLife, 2020. N = 126, 136, and 150 neurons for FEF, caudate, and STN, respectively. Figure 4-S1: Stability of clustering results A, Silhouette scores for different combinations of distance metrics and number of clusters. Red triangle indicates the clustering presented in Figure 4. Mean and s.d. values were computed from 50 iterations of clustering. B, Fraction of negative silhouette scores. Same format as A. C, Rand index. Same format as A. D, Visualization of clusters from Figure 4 in the tSNE space calculated from all units. Non-gray circles indicate neurons that passed visual inspection. Colors indicate cluster identities that are used in Figure 4. E, Average firing rates for each cluster of neurons, wi [file NIHPP2025.11.04.686631v1-supplement-1.pdf]

bioRxiv preprint doi: https://doi.org/10.1101/2025.11.04.686631; this version posted November 6, 2025. The copyright holder for this preprint (which was not certified by peer review) is the author/funder, who has granted bioRxiv a license to display the preprint in perpetuity. It is made available under aCC-BY 4.0 International license.

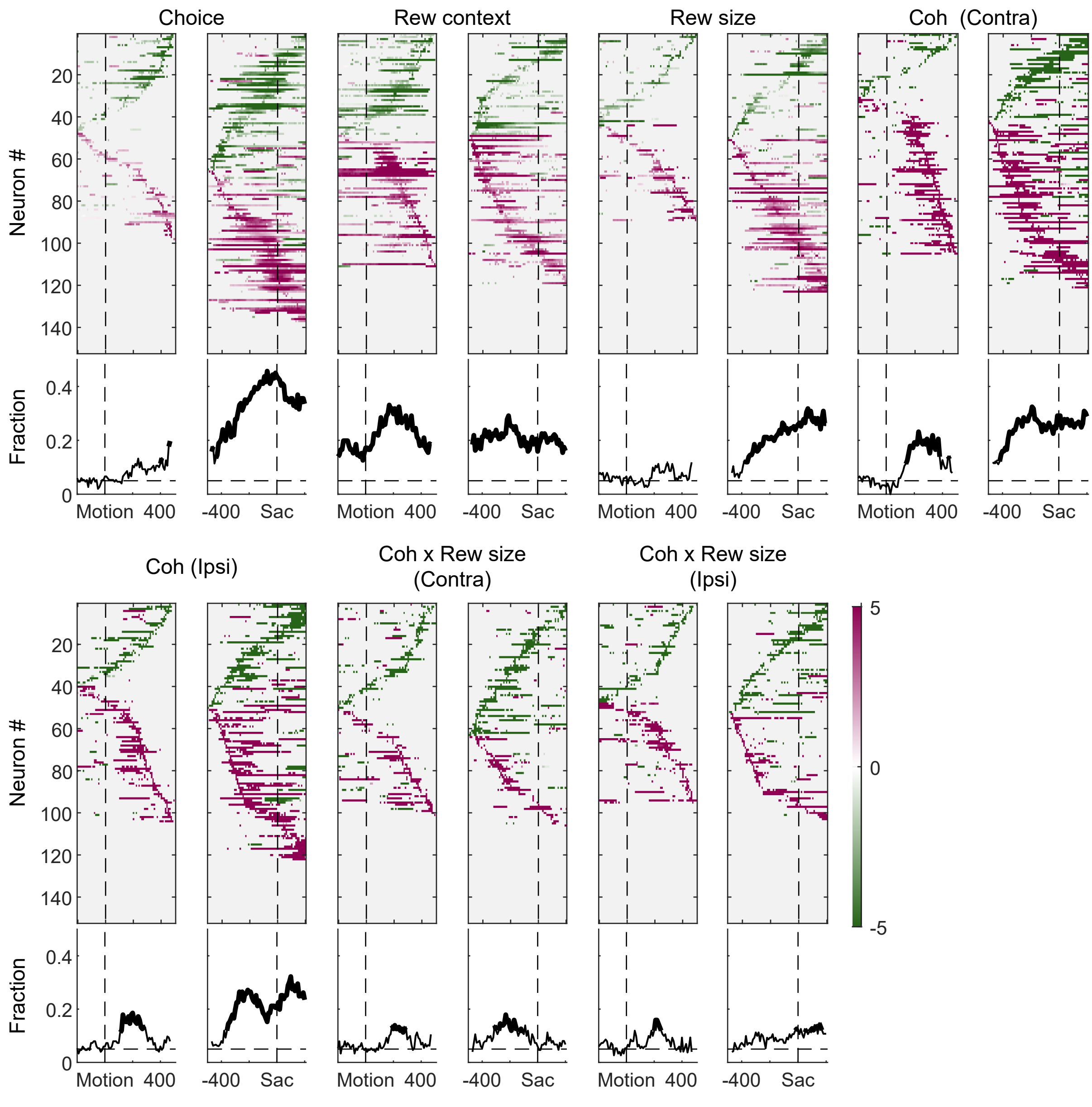

Figure 3-S1: Summary of temporal profiles of modulation of STN activity by decision-related factors.
For each regression factor, heatmaps of significant regression coefficients are plotted in the first row for activity aligned to motion (left) and saccade (right) onsets. Neurons were sorted by the timing of peak magnitude of modulation. The fraction of neurons with significant non-zero coefficients were plotted in the second row. Significance for a coefficient was assessed using t-test (p<0.05). For the fraction plots, the dashed horizontal lines represent chance level. Time bins with values that were significantly above chance (chi-square test, p<0.05) were indicated via thicker lines.

bioRxiv preprint doi: https://doi.org/10.1101/2025.11.04.686631; this version posted November 6, 2025. The copyright holder for this preprint (which was not certified by peer review) is the author/funder, who has granted bioRxiv a license to display the preprint in perpetuity. It is made available under aCC-BY 4.0 International license.

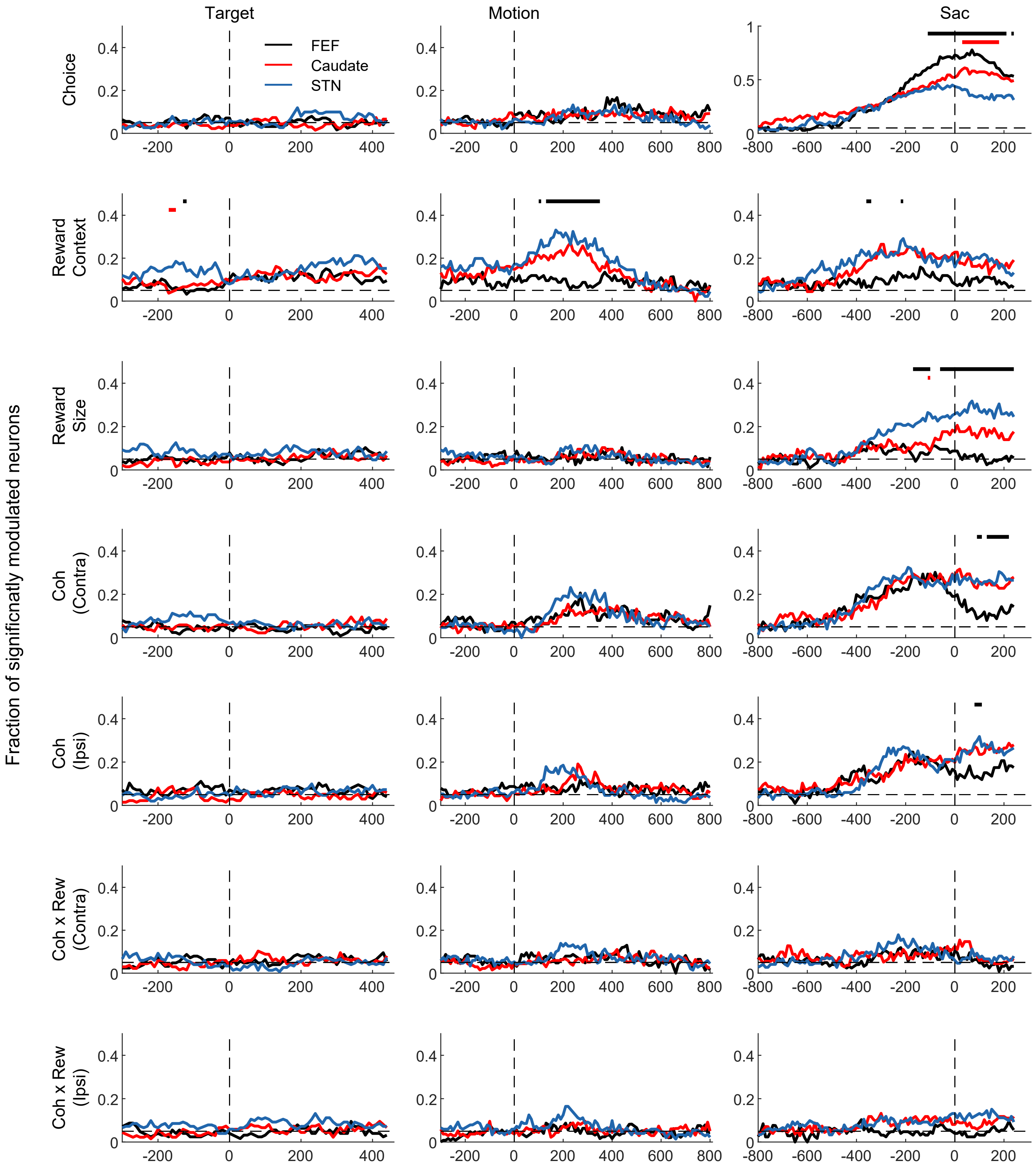

Figure 3-S2: Comparison between STN, FEF and caudate neurons

Each panel shows the fractions of neurons in the three regions (see legend for colors) with significant coefficients (t-test, $p<0.05$) for a specific regressor (rows) and activity alignment (columns). Horizontal bars indicate results of chi-square tests using a criterion of $p<0.05/3$(alignments)/7(regressors). Black horizontal bars indicate significant difference between FEF and STN populations. Red horizontal bars indicate significant difference between caudate and STN populations. FEF and caudate data are from Fan, et al. eLife, 2020. N = 126, 136, and 150 neurons for FEF, caudate, and STN, respectively.

bioRxiv preprint doi: https://doi.org/10.1101/2025.11.04.686631; this version posted November 6, 2025. The copyright holder for this preprint (which was not certified by peer review) is the author/funder, who has granted bioRxiv a license to display the preprint in perpetuity. It is made available under aCC-BY 4.0 International license.

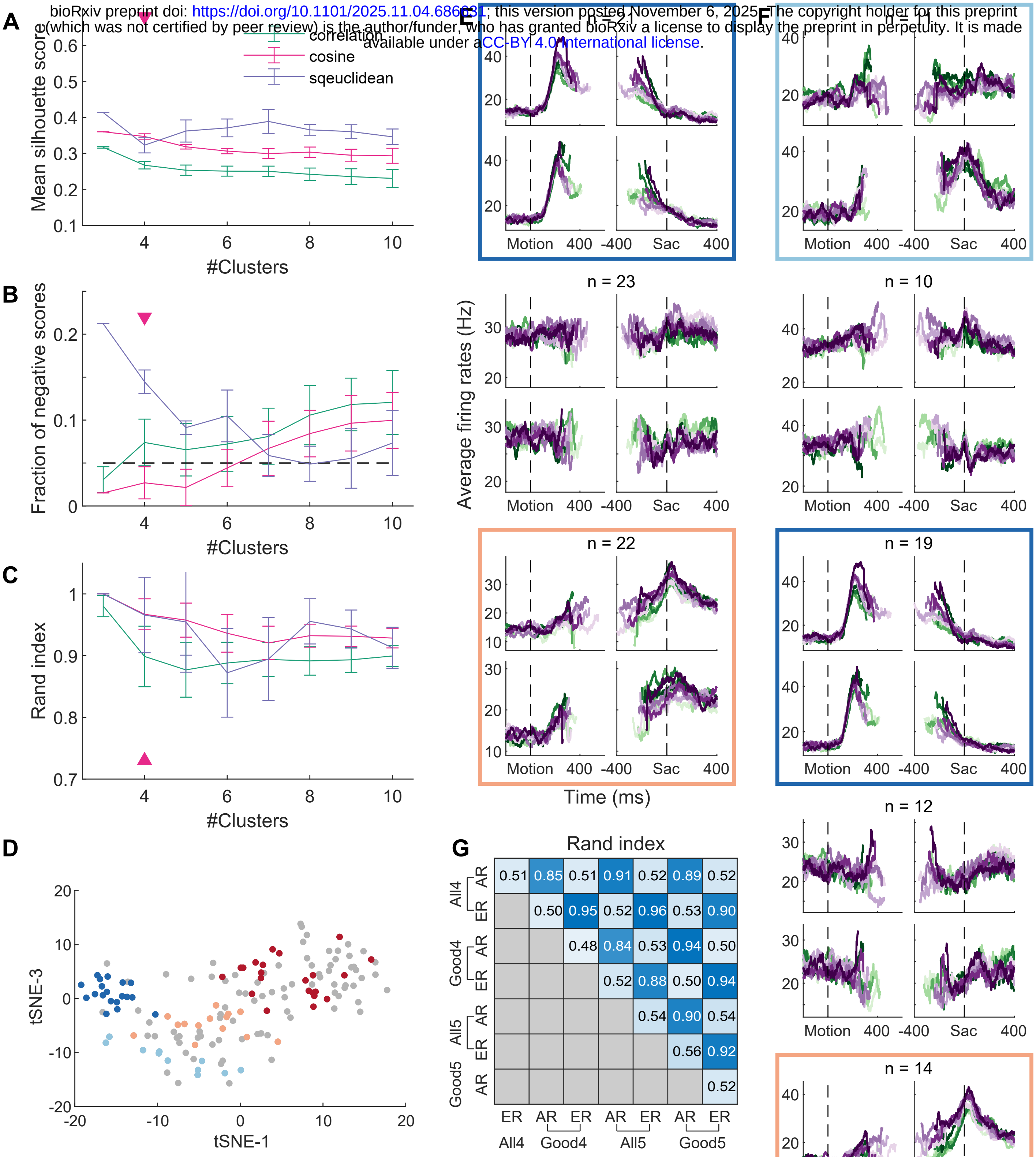

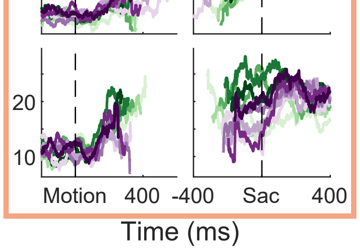

Figure 4-S1: Stability of clustering results

A, Silhouette scores for different combinations of distance metrics and number of clusters. Red triangle indicates the clustering presented in Figure 4. Mean and s.d. values were computed from 50 iterations of clustering.

B, Fraction of negative silhouette scores. Same format as A.

C, Rand index. Same format as A.

D, Visualization of clusters from Figure 4 in the tSNE space calculated from all units. Non-gray circles indicate neurons that passed visual inspection. Colors indicate cluster identities that are used in Figure 4.

E, Average firing rates for each cluster of neurons, with the assumption of three clusters. Same format as Figure 4D. Colored boxes indicate clusters that loosely correspond to those in Figure 4.

F, Average firing rates for each cluster of neurons, with the assumption of five clusters. Same format as Figure E.

G, Pairwise Rand index values for clusterings based on all or only good units, using asymmetric (AR) or equal-reward (ER) data, and assuming 4 or 5 clusters.

bioRxiv preprint doi: https://doi.org/10.1101/2025.11.04.686631; this version posted November 6, 2025. The copyright holder for this preprint (which was not certified by peer review) is the author/funder, who has granted bioRxiv a license to display the preprint in perpetuity. It is made available under aCC-BY 4.0 International license.

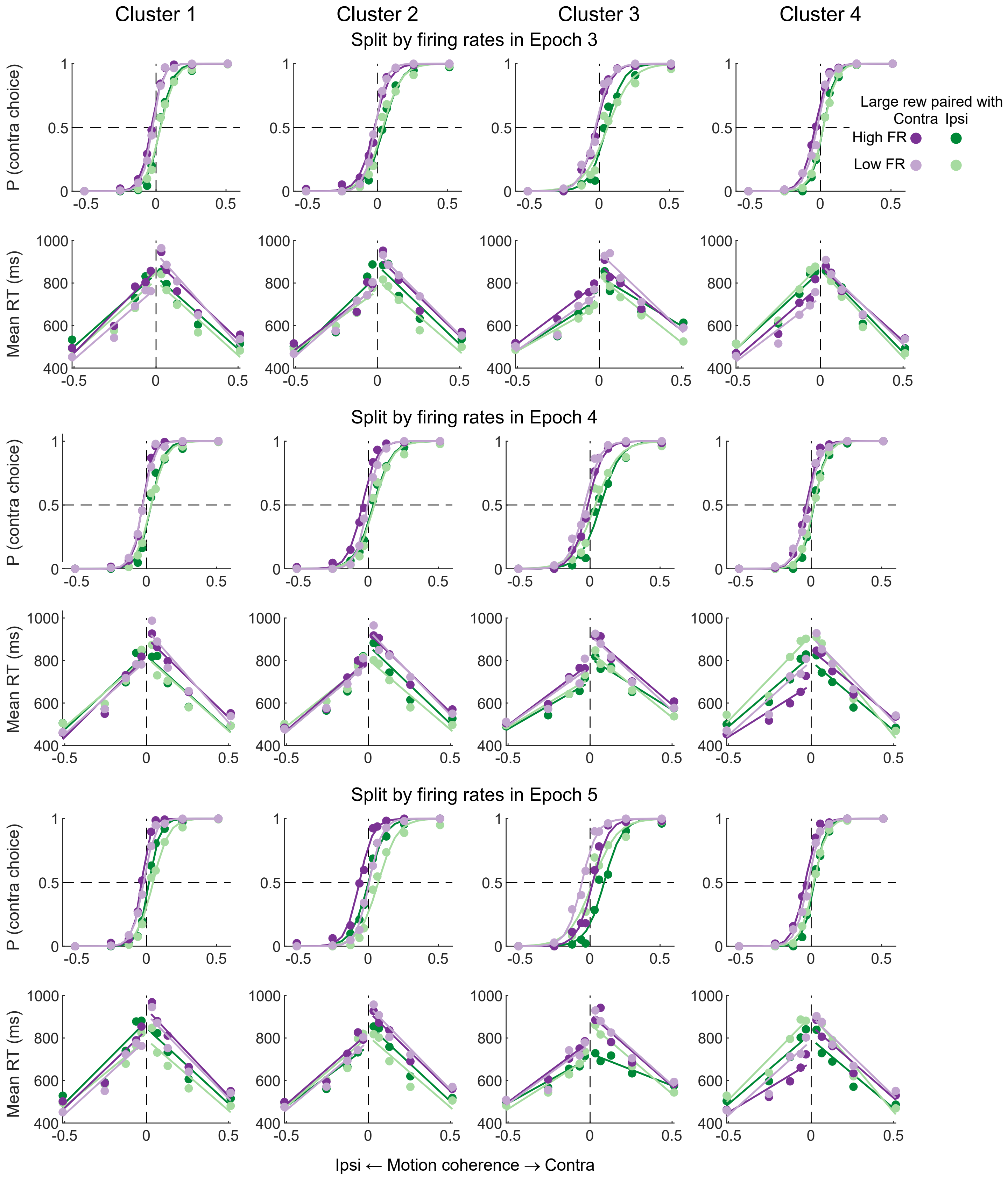

Figure 5 S1 Average choice and RT performance for trials split by firing rates for different neuron clusters and epochs.

Same format as Figure 5B. The columns correspond to the same clusters in Figure 5E.

bioRxiv preprint doi: https://doi.org/10.1101/2025.11.04.686631; this version posted November 6, 2025. The copyright holder for this preprint (which was not certified by peer review) is the author/funder, who has granted bioRxiv a license to display the preprint in perpetuity. It is made available under aCC-BY 4.0 International license.

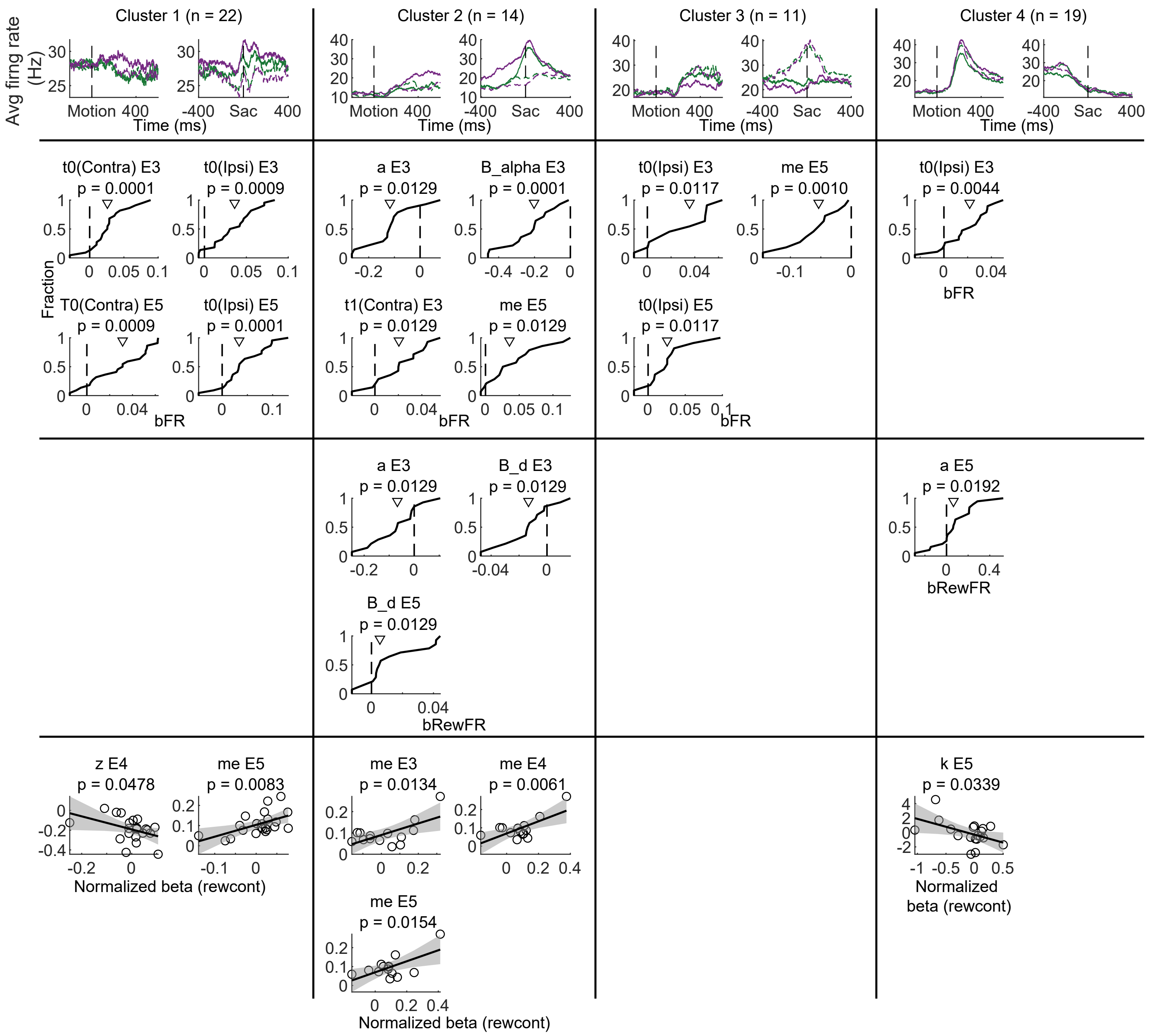

Figure 5 S2 Raw plots corresponding to the summary in Figure 5E

Top section: average firing rates for the four clusters. Same as the top row of Figure 5E.

Second section: Cumulative density functions of regression coefficient (bFR) for DDM parameters. The title for each plot indicates the DDM parameter and the epoch from which firing rates were used to split the trials. Same format as Figure 5C.

Third section: Cumulative density functions of regression coefficient (bRewFR). Same format as the second section.

Bottom section: Scatterplots of the difference in DDM parameters between reward contexts and the regression coefficient for reward context of neural activity. Same format as Figure 5D.

For the last three sections, only relationships with a p<0.05 from sign test (for bFR and bRewFR) or significant Pearson correlation are shown, corresponding to the colored pixels in Figure 5E.

bioRxiv preprint doi: https://doi.org/10.1101/2025.11.04.686631; this version posted November 6, 2025. The copyright holder for this preprint (which was not certified by peer review) is the author/funder, who has granted bioRxiv a license to display the preprint in perpetuity. It is made available under aCC-BY 4.0 International license.

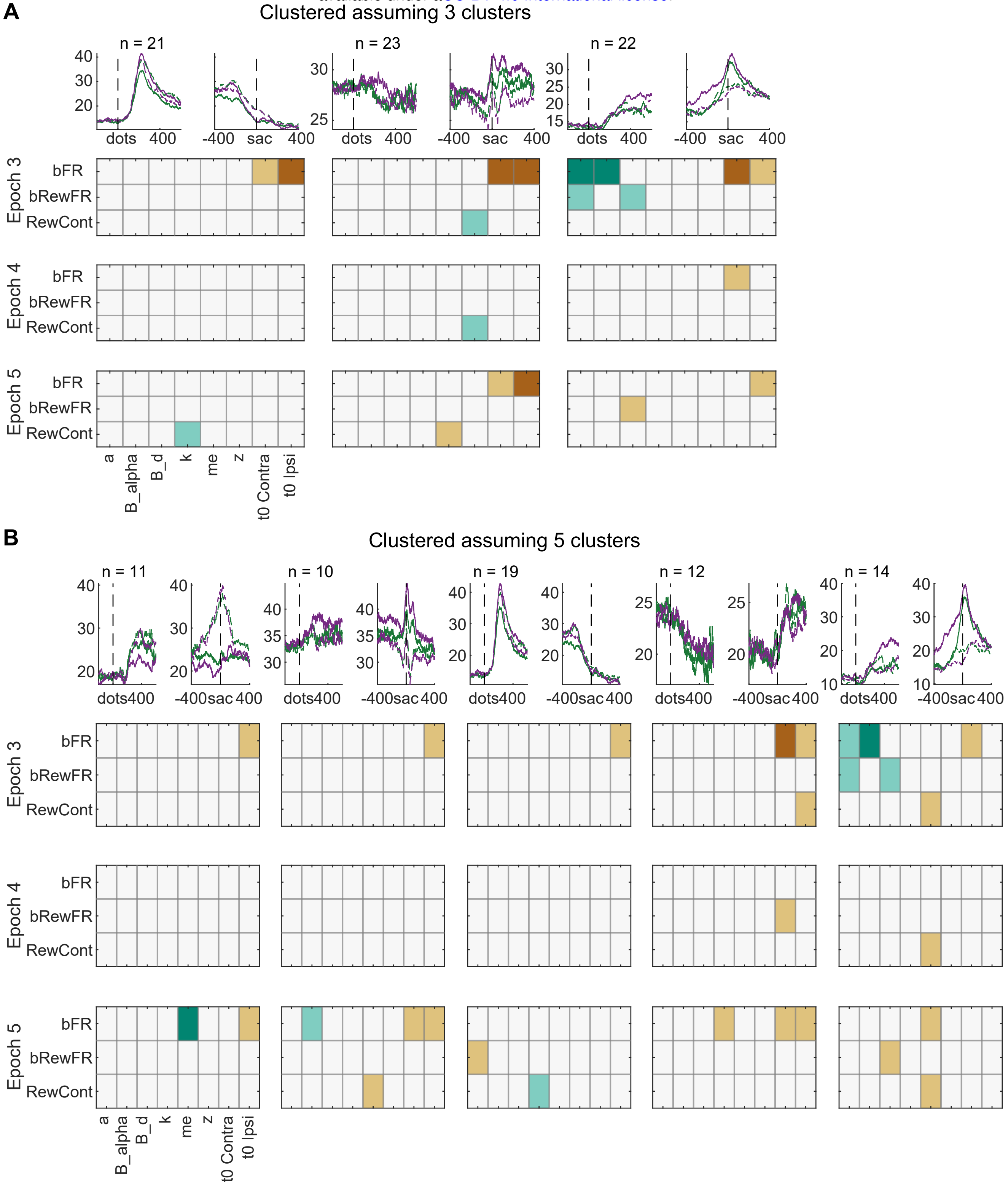

Figure 5 S3 Assessing the robustness of the regression and correlation results with different clustering settings.

A, Regression and correlation results based on dividing the neurons into three clusters. Same format as Figure 5E.

B, Regression and correlation results based on dividing the neurons into five clusters. Same format as Figure 5E.
